# Should colorectal cancer screening start at different ages for men and women? Cost‐effectiveness analysis for a resource‐constrained service

**DOI:** 10.1002/cnr2.1344

**Published:** 2021-02-02

**Authors:** Chloe Thomas, Olena Mandrik, Sophie Whyte, Catherine L. Saunders, Simon J. Griffin, Juliet A. Usher‐Smith

**Affiliations:** ^1^ School of Health and Related Research University of Sheffield Sheffield UK; ^2^ The Primary Care Unit, Department of Public Health and Primary Care University of Cambridge, School of Clinical Medicine Cambridge UK

**Keywords:** cancer screening, colorectal cancer, cost‐effectiveness, fecal immunochemical test, health economic modeling

## Abstract

**Background:**

Men have a greater risk of colorectal cancer (CRC) than women, but population screening currently starts at the same age for both sexes.

**Aim:**

This analysis investigates whether, in a resource‐constrained setting, it would be more effective and cost‐effective for men and women to start screening for CRC at different ages.

**Methods and results:**

An economic modeling analysis was carried out using the Microsimulation Model in Cancer of the Bowel to compare sex‐stratification against screening everyone from the same age, taking an English National Health Service perspective. Screening men from age 56 and women from age 60, rather than screening everyone from age 58 using a Fecal Immunochemical Test (FIT) threshold of 120 μg/g is expected to produce an additional 0.0004 QALYs for a cost of £0.55 per person at model start (Incremental Cost‐effectiveness Ratio = £1392), and to reduce CRC cases and mortality by 25 and 19 per 100 000 people respectively, while using a similar amount of screening resources. Probabilistic sensitivity analysis indicates a 61% probability that sex‐stratification is more cost‐effective than screening everyone at age 58. Similar benefits of sex‐stratification are found at other FIT thresholds, but become negligible if mean screening start age is reduced to 50.

**Conclusion:**

Where resources are constrained and it is not feasible to screen everyone from the age of 50, starting screening earlier in men than women is likely to be more cost‐effective and gain more health benefits overall than strategies where men and women start screening at the same age.

## 1 INTRODUCTION

Colorectal cancer (CRC) is the third most common cancer in men and the second in women worldwide.^1^ There is good evidence that population‐wide screening reduces both CRC incidence and mortality,^2^ and is cost‐effective compared with no screening, irrespective of the screening modality used.^3^ These benefits of screening, together with the increase in incidence of CRC among younger individuals,^4^ have led many countries to reduce the starting age of screening. For example, the UK National Screening Committee has recommended to reduce the start age of biennial screening with fecal immunochemical testing (FIT) from 60 years (as it is currently in England) to 50 years.^5^ However, the incidence of CRC and CRC‐related mortality is higher in men than women^6^: global incidence of CRC is higher in males (746 298 vs 614 304 annually) across all WHO regions and similar trends are noted for CRC‐related mortality with a higher global burden in males (373 639 vs 320 294 annually). Additionally, the location of cancer within the bowel differs between the sexes^7^, ^8^ and CRC occurs at an earlier age in men.^8^, ^9^ The performance of screening tests also differs between men and women: for example, fecal immunochemical testing (FIT) has better performance characteristics with a higher sensitivity for advanced neoplasia and positive predictive value, and a lower specificity and false positive rate in men than women.^10^, ^11^, ^12^ Furthermore, men are less likely to take up screening than women, thus exacerbating inequalities.^13^

These differences have led to proposals that CRC screening should be stratified by sex.^9^, ^14^, ^15^, ^16^ However, worldwide no current CRC screening guidelines include sex‐specific recommendations.^17^ Most previous cost‐effectiveness analyses of screening have also not considered men and women separately.^18^ The only study that considered men and women separately used sex‐specific versions of the MISCAN‐Colon model. That study showed that optimal screening strategies were similar in men and women with respect to interval, age range, and FIT cutoff and although for strategies with few screening rounds sex‐stratified screening dominated uniform screening for both sexes, the differences were small.^19^ However, that study did not consider constraints on resources that operate in practice, with optimal strategies comprising of intensive screening using lower FIT thresholds and lower starting ages than are commonly used worldwide.^20^ Furthermore, differences in uptake between men and women were not considered, but these have important implications for efficacy of screening programs in practice. In order to inform the planned reduction in starting age of screening in England and other countries, we aimed to investigate whether, in a situation of constrained resources, planned reductions in FIT screening start age should be uniform across the population, or stratified by sex.

## 2 MATERIALS AND METHODS

### 2.1 Model background

This analysis uses an individual patient‐level microsimulation model: Microsimulation Model in Cancer of the Bowel (MiMiC‐Bowel) developed in 2019. A detailed description of modeling methods can be found online.^21^ The model has a lifetime horizon and takes an English NHS perspective. Patient baseline characteristics are taken from the Health Survey for England (2014), in order to represent the population of England.^22^ All patients are assumed to be age 30 with normal colorectal epithelium at model start. MiMiC‐Bowel contains a natural history module with patients moving through up to nine different health states representing normal epithelium, low‐ and high‐risk adenoma, CRC stages A to D, and death from CRC or other causes. Transitions between health states were derived through calibration to find parameter sets that enabled the model to replicate age‐ and sex‐specific differences in CRC incidence and prevalence of adenomas and undiagnosed CRC in the absence of CRC screening. CRC mortality rates were calculated by age, sex, stage at diagnosis, and year from diagnosis, while other cause mortality was based on age and sex only.

In the model patients with CRC may be detected via screening or clinically via symptomatic/chance presentation. Modeled screening procedures are based on the English Bowel Cancer Screening Programme (BCSP), with positive results leading to further investigation by colonoscopy. Patients found to have adenomas undergo polypectomy and British Society of Gastroenterology guidelines are implemented in the model for surveillance following adenoma removal.^23^ Complications of endoscopy include perforation, major bleed, and mortality. The model incorporates sex‐ and age‐specific differences in uptake of screening and follow‐up procedures, and screening sensitivity and specificity. All modeled procedures are assumed to incur costs and resource use, with average CRC treatment costs varying by age, stage at diagnosis, and year from diagnosis. All patients have an individual health‐related quality‐of‐life, which is subject to decrements based on age, CRC diagnosis, and endoscopy complications.

### 2.2 Model analyses

The current FIT screening strategy in England (biennial FIT at a threshold of 120 μg/g [FIT120], age 60‐74; eight screening episodes in total) was chosen as the comparator for incremental cost‐effectiveness analysis. As there are plans for the start age for CRC screening to be reduced, the primary analysis asked the question whether it would be more cost‐effective and beneficial to health to reduce screening start age by 2 years to age 58 in all individuals (nine screening episodes in total for all individuals), or whether instead screening start age should be reduced by 4 years to age 56 in males, and kept at age 60 in females (10 screening episodes for males and 8 in females, approximate average of 9 screening episodes in total). This comparison was chosen for two reasons. Firstly, it was important to keep the total number of screening episodes, and hence screening resource uses approximately the same between intervention and comparator to ensure that any benefits of sex‐stratified screening could be attributed to the stratification itself and not to performing more screening overall. This is necessary because previous work has indicated that lowering screening start age in all individuals is cost‐effective and reduces CRC incidence and mortality, but is currently not feasible in England due to resource constraints.^24^ Secondly, Cancer Research UK incidence data indicate that 10 year cumulative incidence of CRC is approximately the same (0.85%) in males aged 56 and females aged 60.^25^ Consequently, choosing a starting age of 56 in males and 60 in females would mean that, on average, the estimated 10‐year CRC risk would be the same for both sexes when first invited for screening. In order to ascertain whether benefits were due specifically to stratification by sex, a further analysis was done in which a randomly selected 50% of the population were screened from age 56, with the other half screened from age 60.

A set of alternative scenario analyses were then carried out to look at the benefits of sex‐stratification with (a) lower FIT thresholds (FIT80 and FIT20); (b) a younger mean start age (age 54 or age 50); (c) lower FIT threshold and younger mean start age (FIT20 at age 50); (d) a different number of years (2, 4, 6 or 8) between the starting age for screening in men and women; and (e) a different discount rate for costs and QALYs (1.5% and 5%, compared to the 3.5% used in the base case analysis) (Table S1). For all analyses, strategies with the same mean starting age and same mean number of screening episodes were compared. For strategies in which males and females started screening at an even numbered age, all individuals were invited for their last screen at age 74. For strategies in which males and females started screening at an odd numbered age, males were assumed to finish screening at age 73, while females were assumed to finish screening at age 75, which ensured that the mean number of screening episodes was the same as the fixed age comparator (eg, screening men from age 57 to 73 and women from 59 to 75, which is 9 screening episodes each).

We modeled the cost‐effectiveness, health benefits, and resource use for each strategy using probabilistic sensitivity analysis (PSA), to enable parameter uncertainty to be incorporated. Discount rate was set at 3.5% for costs and QALYs unless otherwise stated. Cost‐effectiveness was measured using the incremental cost‐effectiveness ratio (ICER) and net monetary benefit (NMB), assuming a willingness to pay threshold of £20 000 per QALY. Outcomes were collected per person in the whole population and by sex.

## 3 RESULTS

**FIGURE 1:**
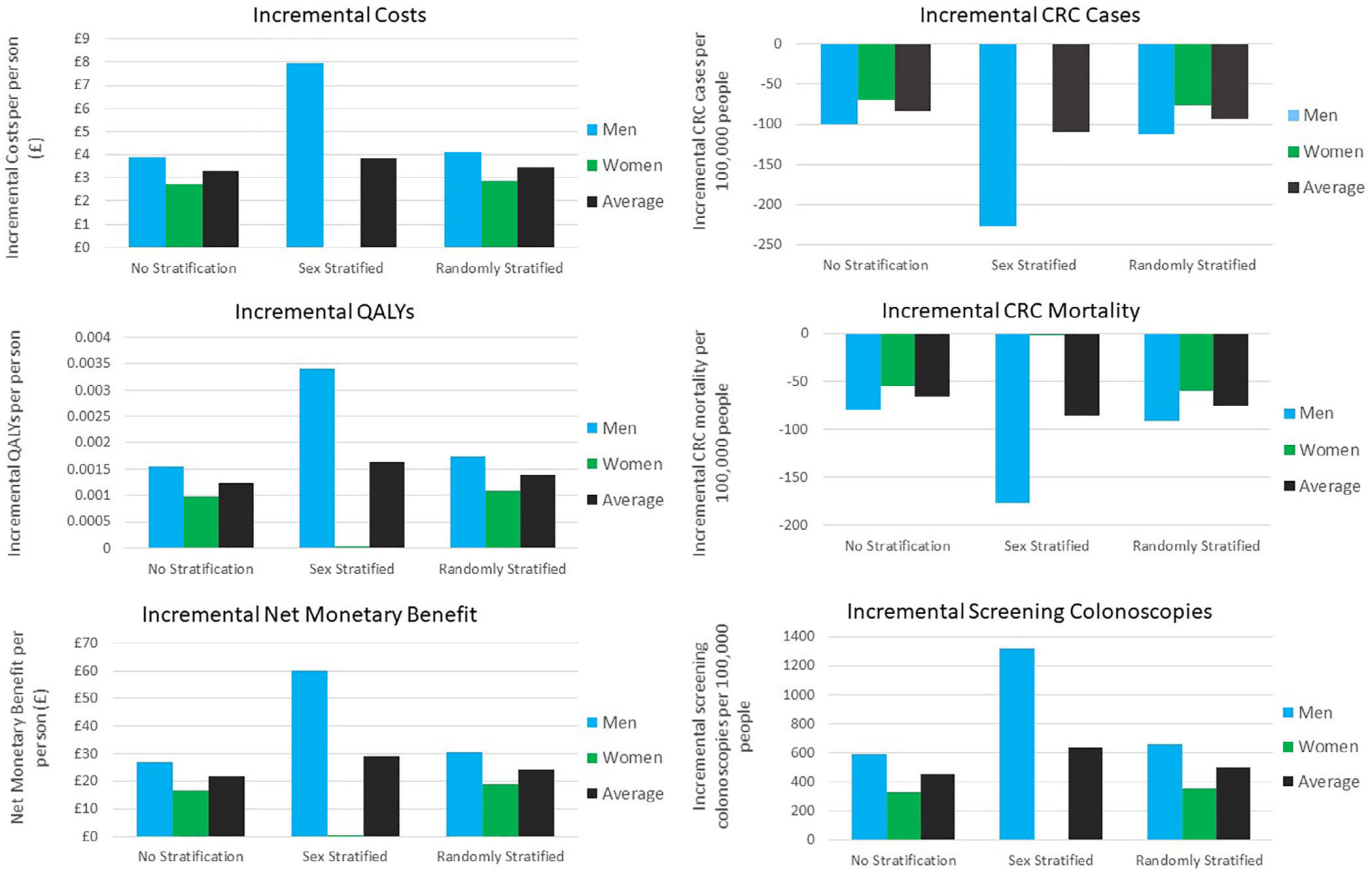
Incremental lifetime cost‐effectiveness outcomes, health benefits, and resource use for men, women, and averaged over the population per person at model start for (A) screening all individuals from age 58 (no stratification); (B) screening men from age 56 and women from age 60 (sex‐stratified); (C) randomly screening half the population from age 56 and half from age 60 (randomly stratified). All incremental results are compared with screening all individuals from age 60 and are based on screening for colorectal cancer (CRC) with the fecal immunochemical test (FIT) at 120 μg/g. Net monetary benefit is calculated by assuming a willingness to pay threshold of £20 000 per quality‐adjusted life year (QALY)

Reducing FIT screening start age from 60 to 58 is cost‐effective (Figure 1 & Table S2), producing 0.0012 QALYs for a cost of £3.28 per person in the population at model start (ICER = £2634). Over a lifetime horizon, the strategy is expected to result in 84 fewer CRC cases, 93 fewer late stage CRC cases, and 66 fewer CRC mortalities per 100 000 population (a 1.3% reduction in CRC incidence and a 2.1% reduction in CRC mortality overall). It is anticipated that this will require an additional 451 screening colonoscopies per 100 000 population compared to current care (an increase of 6.6%). On average, men gain slightly more health benefits from this strategy than women, but also incur greater costs and use slightly more resources, which are explained by their higher underlying risk and hence ability to benefit from screening.

**FIGURE 2:**
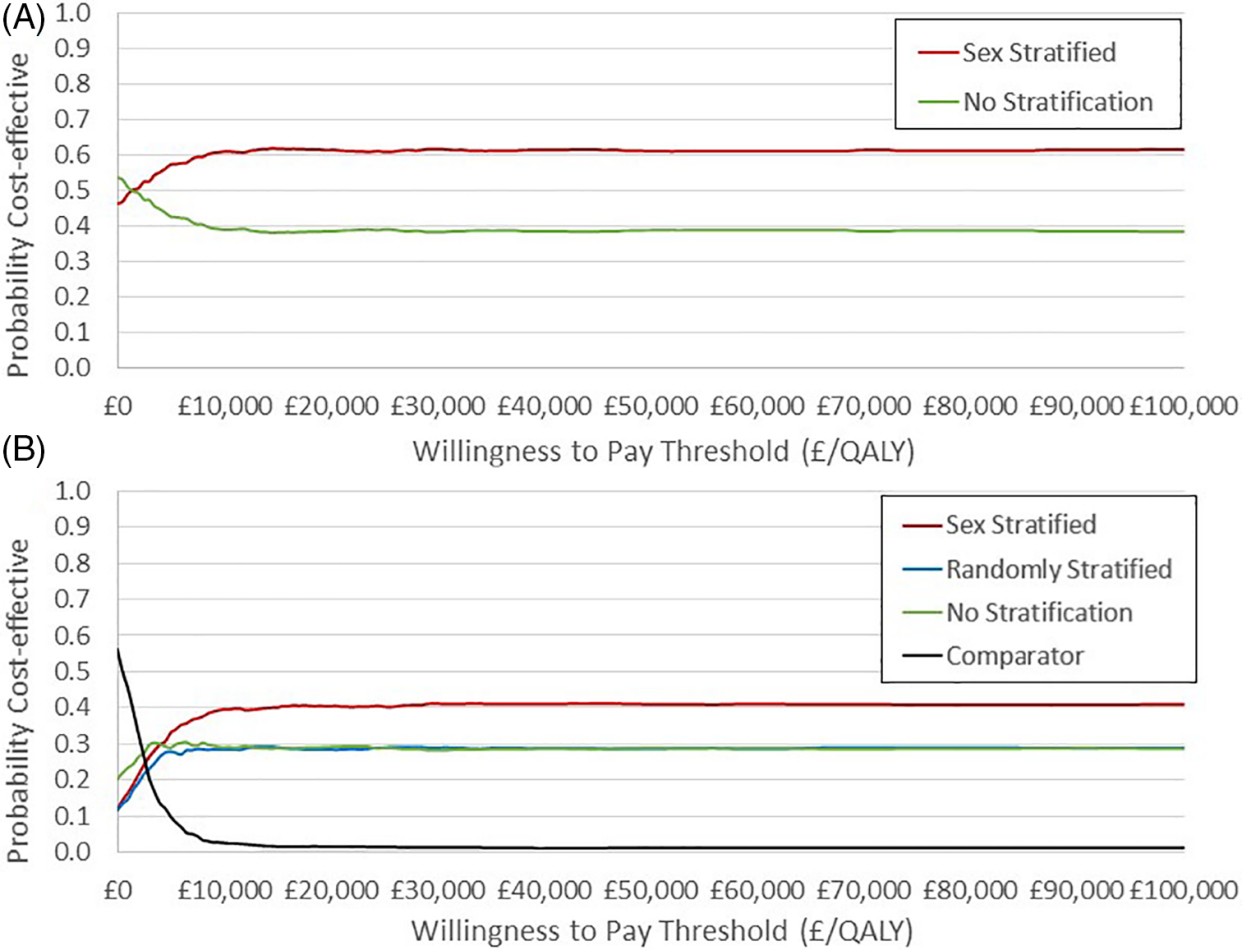
Cost‐effectiveness acceptability curves indicating (a) the probability that sex‐stratified screening (men starting at age 56 and women starting at age 60) is the most cost‐effective option compared with no stratification (screening everyone from age 58); (b) the most cost‐effective strategy when comparing the comparator (screening everyone at age 60), with no stratification (screening everyone at age 58), randomly stratified (half starting at age 56 and half starting at age 60), and sex‐stratified (men starting at age 56 and women starting at age 60); at a range of different willingness to pay thresholds measured in £ per quality‐adjusted life‐year (QALY)

Screening men from age 56 but keeping the screening start age at 60 for women is more cost‐effective than screening everyone from age 58 (Figure 1 & Table S2), producing an additional 0.0004 QALYs for a further cost of only £0.55 per person in the population at model start (ICER = £1392). Probabilistic sensitivity analysis indicates that there is a 61% probability that this sex‐stratified approach is more cost‐effective than screening everyone at age 58, assuming a willingness to pay threshold of £20 000 or £30 000 per QALY (Figure 2). Greater health benefits are also produced, with an additional reduction of 25 CRC cases, 28 late stage CRC cases and 19 CRC mortalities per 100 000 people in the population, compared to screening everyone from 58. As expected, all the additional health benefits are gained by men. This is in the context of similar resource use; slightly fewer FIT tests are required for sex‐stratified screening (due to there being slightly fewer men than women at screening start age) but somewhat more screening colonoscopies are required (a further 185 per 100 000 population), reflecting the higher CRC risk level and hence greater number of positive screening tests in men. Random screening of 50% of the population from age 56 and 50% from age 60 only produces a small fraction of the benefits of sex‐stratified screening, compared with screening everyone from 58 (Figure 1 & Table S2). These small benefits of random stratification compared with screening all at age 58 are likely to be explained by the benefits of half the population starting screening 2 years earlier outweighing the disadvantages of the other half starting screening 2 years later (higher uptake and positivity of screening in the larger younger group).

**FIGURE 3:**
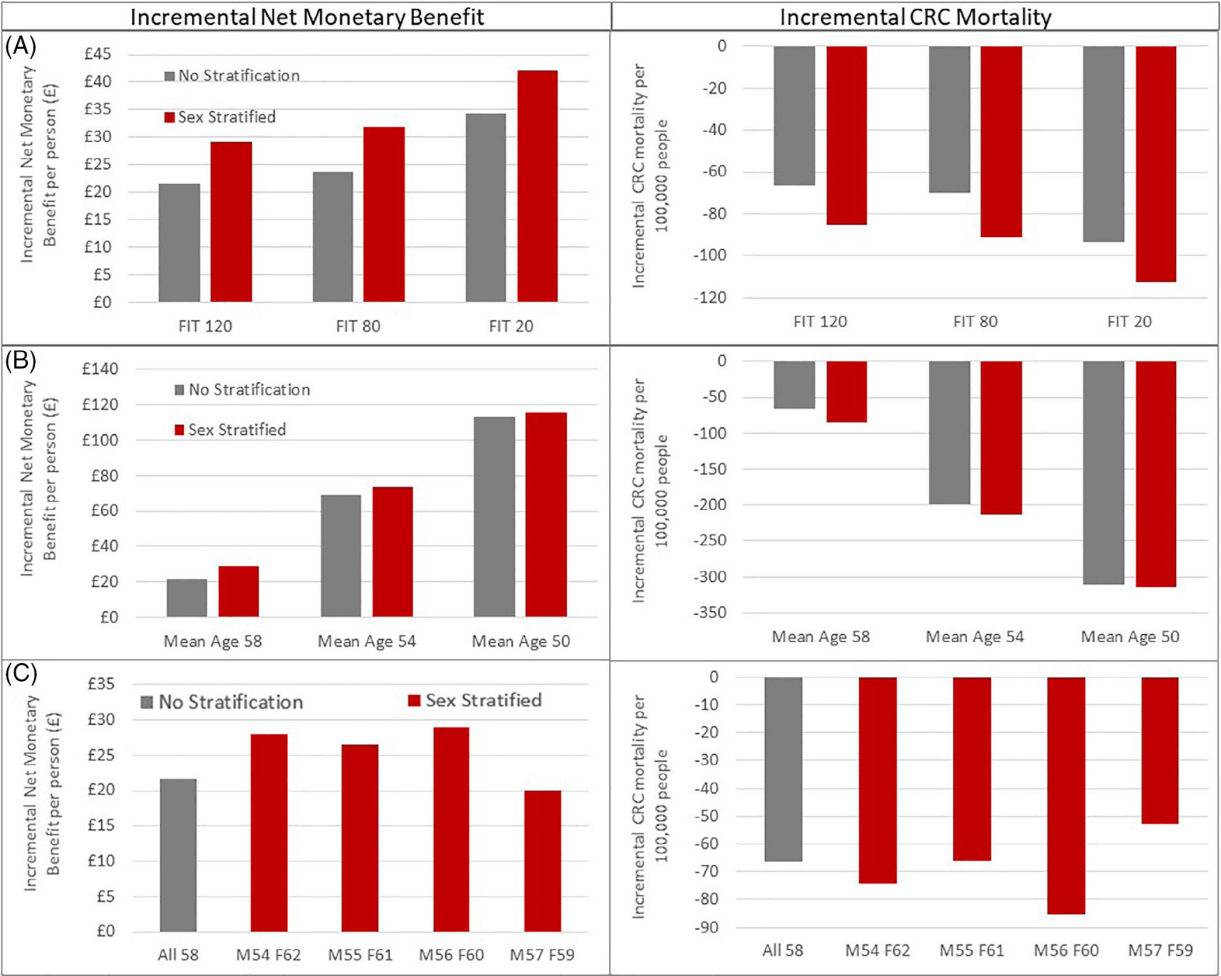
Scenario analysis showing the incremental benefits produced through reducing screening start age without stratification, or with sex stratification at: (A) different fecal immunochemical test (FIT) screening thresholds (in all cases, sex stratification means men screened from age 56 and women screened from age 60); (B) different mean start ages (in all cases, at FIT120 and sex stratification means men screened 4 years earlier than women); (C) different age gaps for screening start by men and women (in all cases, at FIT120 and note that where screening starts at an odd numbered age, it ends at 73 in men and 75 in women to keep mean number of screening episodes constant). All incremental results are compared with screening all individuals from age 60. Net monetary benefit is calculated by assuming a willingness to pay threshold of £20 000 per quality‐adjusted life year (QALY)

Scenario analysis indicates that screening at different FIT thresholds has little impact on the benefits of sex stratification (Figure 3, panel A and Table S3). However, benefits to the population as a whole are considerably smaller if younger mean start age strategies are compared, with there being little difference in overall net monetary benefit or reductions in CRC mortality between screening all individuals from age 50, vs screening men in their late 40s and women in their early 50s (Figure 3, panel B and Table S4). While sex‐stratified screening strategies with an average starting age of 50 years do benefit men more than women, the differences between the sexes are smaller compared to starting screening at age 58 on average, and almost cancel each other out. Similar results are found at a lower FIT threshold (Table S5). The number of years between male and female screening starting age also impacts on the results (Figure 3, panel C and Table S6). Compared with screening everyone at age 58, the optimal sex‐stratified strategy is to screen men at age 56 and women at age 60, which corresponds with differences between the sexes seen in CRC incidence data.^25^ In general, strategies that start at odd‐numbered ages and finish at age 73 for men and age 75 for women are less cost‐effective and less beneficial to health than those where screening starts at even‐numbered ages and both men and women finish screening at age 74. Increasing or reducing the discount rate for costs and QALYs has the expected impact in reducing or increasing (respectively) the magnitude of absolute and incremental health benefits and net monetary benefit produced (Table S7), but does not change the probability of cost‐effectiveness, or the conclusions of the analysis.

## 4 DISCUSSION

Men have a higher risk of CRC than women and currently suffer a greater burden of morbidity and mortality relating to CRC; however, no current screening programs differ in their criteria for men and women. This analysis shows that if resources are not available to screen all individuals from a young age, starting screening at a younger age for men than women would not only go towards reducing the disproportional disease burden in men, but is also likely to be more cost‐effective and gain more health benefits overall than strategies with equivalent number of screening episodes and similar resource use, in which men and women start screening at the same age. It, therefore, represents a way of improving screening efficiency and inequalities between the sexes in CRC outcomes without incurring substantial additional resource use or costs.

If resources become available to enable all individuals to be screened at a younger age, the improvements in efficiency of sex stratification diminish, becoming marginal if everyone is screened from age 50, no matter which FIT threshold is chosen. This result supports the findings of the MISCAN modeling,^19^ which did not find a significant benefit for sex‐stratified screening strategies when considering very high resource use scenarios including low FIT thresholds, young starting ages, and shorter screening intervals. Our findings show that there are likely to be significant benefits to sex stratification in a resource‐constrained situation.

Basing the starting age of screening on sex would be logistically easy to implement, and incur no additional costs outside those required for following up additional positive test results. It may, therefore, be attractive to policy makers both in countries with existing screening programs, such as England, where endoscopy capacity is limited, and in countries yet to introduce population‐wide screening or facing additional resource constraints following the covid‐19 pandemic. In many countries, there is also already a precedent for considering men and women differently within national screening programs. In England, for example, breast and cervical cancer screening is carried out only in women. Research is needed to assess the public acceptability of varying the starting age for screening based on sex.

This analysis used a model with a population representative of England, and that takes into account sex differences at almost all stages of the CRC natural history and screening pathways, and therefore is likely to provide a fairly accurate estimate of how the benefits of CRC screening differ by sex. However, model uncertainty is high due to wide confidence intervals around many of the model parameters. These include FIT screening characteristics, which are derived from the relatively small numbers in the UK FIT pilot (FIT was only rolled out from mid‐2019 in England), the utility decrements for people diagnosed with CRC, and uncertainties around the data used to calibrate the model natural history, much of which is based on the old Dukes staging system rather than the now more commonly used TNM system. Improvements in data collection from CRC patients should enable modeled uncertainty to be reduced in future analyses.

## CONFLICT OF INTEREST

The authors declare no conflict of interest.

## AUTHOR CONTRIBUTIONS

C.T. co‐designed the study, co‐developed the model, designed and set up model runs, analyzed and interpreted model results, and co‐wrote the manuscript. She is the guarantor. O.M. co‐developed the model, interpreted the results, obtained funding to carry out modeling analyses, and revised the draft manuscript. S.W. obtained funding to develop the model, contributed to study design, interpreted the results, and revised the draft manuscript. C.L.S. interpreted the results and revised the draft manuscript. S.J.G. interpreted the results and revised the draft manuscript. J.A.U.S. co‐designed the study, obtained funding to support model development, interpreted the results, and co‐wrote the manuscript. The corresponding author attests that all listed authors meet authorship criteria and that no others meeting the criteria have been omitted.

## ETHICAL APPROVAL AND CONSENT TO PARTICIPATE

Ethical approval was not required for this work as it is based only on published data.

## Supporting information

**Appendix S1.** Supporting information.

## Data Availability

No data collection was carried out as part of this study.
