# Supplementary material for: Should colorectal cancer screening start at different ages for men and women? Cost‐effectiveness analysis for a resource‐constrained service
Source: Cancer Rep (Hoboken). 2021 Feb 2;4(4):e1344. doi: 10.1002/cnr2.1344 (PMC8388164; doi:10.1002/cnr2.1344)
Supplement: Supplementary file 1 — Appendix S1. Supporting information. [file CNR2-4-e1344-s001.docx]

Supplementary Table 1: A summary of the modelled screening strategies.

| **Screening Strategy** | **Screening Start Age** | | | **Screening End Age** | | | **Number Screening Episodes** | | | **FIT Threshold (µg/g)** | **Discount Rate** |
| --- | --- | --- | --- | --- | --- | --- | --- | --- | --- | --- | --- |
|  | Male | Female | Mean | Male | Female | Mean | Male | Female | Mean |  |  |
| **Primary Analysis** | | | | | | | | | | | |
| All 60 (comparator) | 60 | 60 | 60 | 74 | 74 | 74 | 8 | 8 | 8 | 120 | 3.5% |
| All 58 | 58 | 58 | 58 | 74 | 74 | 74 | 9 | 9 | 9 | 120 | 3.5% |
| M56 F60 | 56 | 60 | 58 | 74 | 74 | 74 | 10 | 8 | 9 | 120 | 3.5% |
| Random 56_60 | 58* | 58* | 58 | 74 | 74 | 74 | 9* | 9* | 9 | 120 | 3.5% |
| **Scenario A: Alternative FIT Thresholds** | | | | | | | | | | | |
| FIT80 All 60 (comparator) | 60 | 60 | 60 | 74 | 74 | 74 | 8 | 8 | 8 | 80 | 3.5% |
| FIT80 All 58 | 58 | 58 | 58 | 74 | 74 | 74 | 9 | 9 | 9 | 80 | 3.5% |
| FIT80 M56 F60 | 56 | 60 | 58 | 74 | 74 | 74 | 10 | 8 | 9 | 80 | 3.5% |
| FIT20 All 60 (comparator) | 60 | 60 | 60 | 74 | 74 | 74 | 8 | 8 | 8 | 20 | 3.5% |
| FIT20 All 58 | 58 | 58 | 58 | 74 | 74 | 74 | 9 | 9 | 9 | 20 | 3.5% |
| FIT20 M56 F60 | 56 | 60 | 58 | 74 | 74 | 74 | 10 | 8 | 9 | 20 | 3.5% |
| **Scenario B: Alternative Mean Screening Start Age** | | | | | | | | | | | |
| All 54 | 54 | 54 | 54 | 74 | 74 | 74 | 11 | 11 | 11 | 120 | 3.5% |
| M52 F56 | 52 | 56 | 54 | 74 | 74 | 74 | 12 | 10 | 11 | 120 | 3.5% |
| All 50 | 50 | 50 | 50 | 74 | 74 | 74 | 13 | 13 | 13 | 120 | 3.5% |
| M48 F52 | 48 | 52 | 50 | 74 | 74 | 74 | 14 | 12 | 13 | 120 | 3.5% |
| **Scenario C: Alternative FIT Thresholds and Mean Screening Start Age** | | | | | | | | | | | |
| FIT 20 All 50 | 50 | 50 | 50 | 74 | 74 | 74 | 13 | 13 | 13 | 20 | 3.5% |
| FIT20 M48 F52 | 48 | 52 | 50 | 74 | 74 | 74 | 14 | 12 | 13 | 20 | 3.5% |
| **Scenario D: Alternative Numbers of Years Between Male and Female Screening Start Age** | | | | | | | | | | | |
| M54 F62 | 54 | 62 | 58 | 74 | 74 | 74 | 11 | 7 | 9 | 120 | 3.5% |
| M55 F61 | 55 | 61 | 58 | 73 | 75 | 74 | 10 | 8 | 9 | 120 | 3.5% |
| M57 F59 | 57 | 59 | 58 | 73 | 75 | 74 | 9 | 9 | 9 | 120 | 3.5% |
| **Scenario E: Alternative Discount Rates** | | | | | | | | | | | |
| All 60 DR1.5% (comparator) | 60 | 60 | 60 | 74 | 74 | 74 | 8 | 8 | 8 | 120 | 1.5% |
| All 58 DR1.5% | 58 | 58 | 58 | 74 | 74 | 74 | 9 | 9 | 9 | 120 | 1.5% |
| M56 F60 DR1.5% | 56 | 60 | 58 | 74 | 74 | 74 | 10 | 8 | 9 | 120 | 1.5% |
| All 60 DR5% (comparator) | 60 | 60 | 60 | 74 | 74 | 74 | 8 | 8 | 8 | 120 | 5.0% |
| All 58 DR5% | 58 | 58 | 58 | 74 | 74 | 74 | 9 | 9 | 9 | 120 | 5.0% |
| M56 F60 DR5% | 56 | 60 | 58 | 74 | 74 | 74 | 10 | 8 | 9 | 120 | 5.0% |
| FIT = faecal immunochemical test. *on average. | | | | | | | | | | | |

Supplementary Table 2: Absolute and incremental average outcomes for cost-effectiveness, health benefits and resource use over the lifetime of each person in the English population at model start (age 30), comparing starting faecal immunochemical test (FIT) screening at a threshold of 120µg/g in all individuals at age 60 (comparator) against starting screening in all individuals at age 58 (no stratification), starting screening in men at age 56 and women at age 60 (sex stratified) or starting screening in half the population at age 56 and half at 60 (randomly stratified).

|  | **Absolute Values** | | | | **Incremental vs Comparator** | | | **Incremental vs No Stratification** | |
| --- | --- | --- | --- | --- | --- | --- | --- | --- | --- |
|  | **Comparator**  (all start age 60) | N**o Stratification** (all start age 58) | **Sex Stratified** (men start age 56, women start age 60) | **Randomly Stratified** (half start age 56, half start age 60) | **No Stratification**  (all start age 58) | **Sex Stratified** (men start age 56, women start age 60) | **Randomly Stratified** (half start age 56, half start age 60) | **Sex Stratified** (men start age 56, women start age 60) | **Randomly Stratified** (half start age 56, half start age 60) |
| Lifetime Costs (£ per person, discounted) | £459.50 | £462.78 | £463.33 | £462.96 | £3.28 | £3.83 | £3.46 | £0.55 | £0.18 |
| Lifetime QALYs (per person, discounted) | 19.8274 | 19.8286 | 19.8290 | 19.8288 | 0.0012 | 0.0016 | 0.0014 | 0.0004 | 0.0001 |
| Lifetime NMB (per person, £20,000 per QALY) | £396,088 | £396,110 | £396,117 | £396,113 | £22 | £29 | £24 | £7 | £3 |
| Lifetime NMB (per person, £30,000 per QALY) | £594,362 | £594,396 | £594,408 | £594,401 | £34 | £45 | £38 | £11 | £4 |
| ICER (£ per QALY) | NA | NA | NA | NA | £2,634 | £2,334 | £2,483 | £1,392 | £1,212 |
| Late Stage CRC (per 100,000 people) | 4182 | 4088 | 4060 | 4078 | -93 | -122 | -103 | -28 | -10 |
| Late Stage CRC Incidence Reduction (%) | NA | NA | NA | NA | 2.2% | 2.9% | 2.5% | 0.7% | 0.2% |
| Total CRC (per 100,000 people) | 6605 | 6520 | 6496 | 6511 | -84 | -109 | -94 | -25 | -9 |
| Total CRC Incidence Reduction (%) | NA | NA | NA | NA | 1.3% | 1.7% | 1.4% | 0.4% | 0.1% |
| CRC Mortality (per 100,000 people) | 3121 | 3055 | 3036 | 3046 | -66 | -85 | -75 | -19 | -8 |
| CRC Mortality Reduction (%) | NA | NA | NA | NA | 2.1% | 2.7% | 2.4% | 0.6% | 0.3% |
| FIT Invites (per person) | 6.72 | 7.64 | 7.60 | 7.65 | 0.92 | 0.88 | 0.93 | -0.04 | 0.00 |
| FIT Responses (per person) | 4.88 | 5.45 | 5.47 | 5.53 | 0.57 | 0.59 | 0.65 | 0.02 | 0.07 |
| Screening Colonoscopies (per 100,000) | 6855 | 7306 | 7491 | 7355 | 451 | 636 | 500 | 185 | 49 |
| Increase in Colonoscopy Usage (%) | NA | NA | NA | NA | 6.6% | 9.3% | 7.3% | 2.5% | 0.7% |
| QALY Quality Adjusted Life Year; NMB Net Monetary Benefit (calculated assuming a willingness to pay threshold of £20,000 or £30,000 per QALY); ICER Incremental Cost-effectiveness Ratio; CRC Colorectal Cancer; FIT Faecal Immunochemical Test; NA Not Applicable. | | | | | | | | | |

Supplementary Table 3: Full outcomes for scenario A: Alternative FIT thresholds. Absolute and incremental average outcomes for cost-effectiveness, health benefits and resource use over the lifetime of each person in the English population at model start (age 30).

|  | **Absolute Values** | | | | | | **Incremental vs Comparator** | | | |
| --- | --- | --- | --- | --- | --- | --- | --- | --- | --- | --- |
|  | **Comparator FIT80**  (all start age 60) | N**o Stratification FIT80** (all start age 58) | **Sex Stratified FIT80** (men start age 56, women start age 60) | **Comparator FIT20**  (all start age 60) | N**o Stratification FIT20** (all start age 58) | **Sex Stratified FIT20** (men start age 56, women start age 60) | N**o Stratification FIT80** (all start age 58) | **Sex Stratified FIT80** (men start age 56, women start age 60) | N**o Stratification FIT20** (all start age 58) | **Sex Stratified FIT20** (men start age 56, women start age 60) |
| Lifetime Costs (£ per person, discounted) | £457.06 | £459.72 | £460.30 | £453.98 | £455.74 | £456.16 | £2.66 | £3.23 | £1.76 | £2.18 |
| Lifetime QALYs (per person, discounted) | 19.8274 | 19.8287 | 19.8292 | 19.8323 | 19.8341 | 19.8345 | 0.0013 | 0.0018 | 0.0018 | 0.0022 |
| Lifetime NMB (per person, £20,000 per QALY) | £396,092 | £396,115 | £396,123 | £396,192 | £396,227 | £396,234 | £24 | £32 | £34 | £42 |
| Lifetime NMB (per person, £30,000 per QALY) | £594,366 | £594,403 | £594,415 | £594,515 | £594,568 | £594,580 | £37 | £49 | £52 | £64 |
| ICER (£ per QALY) | NA | NA | NA | NA | NA | NA | £2,017 | £1,840 | £974 | £984 |
| Late Stage CRC (per 100,000 people) | 4027 | 3928 | 3896 | 3392 | 3265 | 3233 | -99 | -131 | -126 | -159 |
| Late Stage CRC Incidence Reduction (%) | NA | NA | NA | NA | NA | NA | 2.4% | 3.3% | 3.7% | 4.7% |
| Total CRC (per 100,000 people) | 6403 | 6302 | 6274 | 5534 | 5378 | 5344 | -101 | -129 | -155 | -189 |
| Total CRC Incidence Reduction (%) | NA | NA | NA | NA | NA | NA | 1.6% | 2.0% | 2.8% | 3.4% |
| CRC Mortality (per 100,000 people) | 3003 | 2934 | 2912 | 2505 | 2411 | 2392 | -70 | -91 | -94 | -113 |
| CRC Mortality Reduction (%) | NA | NA | NA | NA | NA | NA | 2.3% | 3.0% | 3.7% | 4.5% |
| FIT Invites (per person) | 6.70 | 7.62 | 7.58 | 6.63 | 7.55 | 7.50 | 0.92 | 0.88 | 0.91 | 0.87 |
| FIT Responses (per person) | 4.87 | 5.44 | 5.45 | 4.80 | 5.37 | 5.38 | 0.57 | 0.59 | 0.56 | 0.58 |
| Screening Colonoscopies (per 100,000) | 8459 | 9001 | 9225 | 18864 | 20002 | 20363 | 542 | 766 | 1138 | 1498 |
| Increase in Colonoscopy Usage (%) | NA | NA | NA | NA | NA | NA | 6.4% | 9.1% | 6.0% | 7.9% |
| QALY Quality Adjusted Life Year; NMB Net Monetary Benefit (calculated assuming a willingness to pay threshold of £20,000 or £30,000 per QALY); ICER Incremental Cost-effectiveness Ratio; CRC Colorectal Cancer; FIT Faecal Immunochemical Test; NA Not Applicable. | | | | | | | | | | |

Supplementary Table 4: Full outcomes for scenario B: Alternative mean screening start age. Absolute and incremental average outcomes for cost-effectiveness, health benefits and resource use over the lifetime of each person in the English population at model start (age 30).

|  | **Absolute Values** | | | | | **Incremental vs Comparator** | | | |
| --- | --- | --- | --- | --- | --- | --- | --- | --- | --- |
|  | **Comparator** (all start age 60) | N**o Stratification** (all start age 54) | **Sex Stratified** (men start age 52, women start age 56) | N**o Stratification** (all start age 50) | **Sex Stratified** (men start age 48, women start age 52) | N**o Stratification** (all start age 54) | **Sex Stratified** (men start age 52, women start age 56) | N**o Stratification** (all start age 50) | **Sex Stratified** (men start age 48, women start age 52) |
| Lifetime Costs (£ per person, discounted) | £459.50 | £468.86 | £469.77 | £476.72 | £477.36 | £9.87 | £9.49 | £17.72 | £18.36 |
| Lifetime QALYs (per person, discounted) | 19.8274 | 19.8293 | 19.8296 | 19.8319 | 19.8321 | 0.0039 | 0.0040 | 0.0066 | 0.0067 |
| Lifetime NMB (per person, £20,000 per QALY) | £396,088 | £396,117 | £396,122 | £396,162 | £396,164 | £69 | £71 | £113 | £116 |
| Lifetime NMB (per person, £30,000 per QALY) | £594,302 | £594,410 | £594,418 | £594,481 | £594,485 | £108 | £112 | £179 | £183 |
| ICER (£ per QALY) | NA | NA | NA | NA | NA | £2,508 | £2,352 | £2,702 | £2,733 |
| Late Stage CRC (per 100,000 people) | 4182 | 3901 | 3883 | 3743 | 3735 | -282 | -301 | -440 | -449 |
| Late Stage CRC Incidence Reduction (%) | NA | NA | NA | NA | NA | 6.8% | 7.2% | 10.5% | 10.7% |
| Total CRC (per 100,000 people) | 6605 | 6336 | 6321 | 6186 | 6182 | -264 | -279 | -414 | -418 |
| Total CRC Incidence Reduction (%) | NA | NA | NA | NA | NA | 4.0% | 4.2% | 6.3% | 6.3% |
| CRC Mortality (per 100,000 people) | 3121 | 2924 | 2911 | 2813 | 2809 | -200 | -213 | -311 | -314 |
| CRC Mortality Reduction (%) | NA | NA | NA | NA | NA | 6.4% | 6.8% | 10.0% | 10.1% |
| FIT Invites (per person) | 6.72 | 9.52 | 9.47 | 11.43 | 11.39 | 2.80 | 2.76 | 4.71 | 4.67 |
| FIT Responses (per person) | 4.88 | 6.81 | 6.80 | 8.28 | 8.23 | 1.93 | 1.92 | 3.40 | 3.35 |
| Screening Colonoscopies (per 100,000) | 6855 | 8239 | 8370 | 9047 | 9125 | 1391 | 1522 | 2199 | 2277 |
| Increase in Colonoscopy Usage (%) | NA | NA | NA | NA | NA | 20.3% | 22.2% | 32.1% | 33.2% |
| QALY Quality Adjusted Life Year; NMB Net Monetary Benefit (calculated assuming a willingness to pay threshold of £20,000 or £30,000 per QALY); ICER Incremental Cost-effectiveness Ratio; CRC Colorectal Cancer; FIT Faecal Immunochemical Test; NA Not Applicable. | | | | | | | | | |

Supplementary Table 5: Full outcomes for scenario C: Alternative FIT threshold and mean screening start age. Absolute and incremental average outcomes for cost-effectiveness, health benefits and resource use over the lifetime of each person in the English population at model start (age 30).

|  | **Absolute Values** | | | **Incremental vs Comparator** | |
| --- | --- | --- | --- | --- | --- |
|  | **Comparator FIT20**  (all start age 60) | N**o Stratification FIT20** (all start age 50) | **Sex Stratified FIT20** (men start age 48, women start age 52) | N**o Stratification FIT20** (all start age 50) | **Sex Stratified FIT20** (men start age 48, women start age 52) |
| Lifetime Costs (£ per person, discounted) | £454.26 | £465.73 | £466.65 | £11.47 | £12.38 |
| Lifetime QALYs (per person, discounted) | 19.8317 | 19.8414 | 19.8415 | 0.0097 | 0.0098 |
| Lifetime NMB (per person) | £396,179 | £396,362 | £396,364 | £183 | £185 |
| Lifetime NMB (per person, £20,000 per QALY) | £594,496 | £594,776 | £594,779 | £280 | £283 |
| Lifetime NMB (per person, £30,000 per QALY) | NA | NA | NA | £1,180 | £1,257 |
| Late Stage CRC (per 100,000 people) | 3405 | 2777 | 2773 | -628 | -632 |
| Late Stage CRC Incidence Reduction (%) | NA | NA | NA | 18.4% | 18.6% |
| Total CRC (per 100,000 people) | 5548 | 4800 | 4798 | -747 | -750 |
| Total CRC Incidence Reduction (%) | NA | NA | NA | 13.5% | 13.5% |
| CRC Mortality (per 100,000 people) | 2513 | 2067 | 2064 | -446 | -448 |
| CRC Mortality Reduction (%) | NA | NA | NA | 17.8% | 17.8% |
| FIT Invites (per person) | 6.63 | 11.29 | 11.25 | 4.66 | 4.62 |
| FIT Responses (per person) | 4.80 | 8.15 | 8.11 | 3.35 | 3.31 |
| Screening Colonoscopies (per 100,000) | 18791 | 24572 | 24696 | 5781 | 5905 |
| Increase in Colonoscopy Usage (%) | NA | NA | NA | 30.8% | 31.4% |
| QALY Quality Adjusted Life Year; NMB Net Monetary Benefit (calculated assuming a willingness to pay threshold of £20,000 or £30,000 per QALY); ICER Incremental Cost-effectiveness Ratio; CRC Colorectal Cancer; FIT Faecal Immunochemical Test; NA Not Applicable. | | | | | |

Supplementary Table 6: Full outcomes for scenario D: Alternative numbers of years between male and female screening start age. Absolute and incremental average outcomes for cost-effectiveness, health benefits and resource use over the lifetime of each person in the English population at model start (age 30).

|  | **Absolute Values** | | | | | **Incremental vs Comparator** | | | |
| --- | --- | --- | --- | --- | --- | --- | --- | --- | --- |
|  | **Comparator** (all start age 60) | N**o Stratification** (all start age 58) | **Sex Stratified** (men start age 54, women start age 62) | **Sex Stratified** (men start age 55, women start age 61) | **Sex Stratified** (men start age 57, women start age 59) | N**o Stratification** (all start age 58) | **Sex Stratified** (men start age 54, women start age 62) | **Sex Stratified** (men start age 55, women start age 61) | **Sex Stratified** (men start age 57, women start age 59) |
| Lifetime Costs (£ per person, discounted) | £459.50 | £462.78 | £463.47 | £462.51 | £461.87 | £3.28 | £3.97 | £3.01 | £2.38 |
| Lifetime QALYs (per person, discounted) | 19.8274 | 19.8286 | 19.8290 | 19.8289 | 19.8285 | 0.0012 | 0.0016 | 0.0015 | 0.0011 |
| Lifetime NMB (per person, £20,000 per QALY) | £396,088 | £396,110 | £396,116 | £396,115 | £396,108 | £22 | £28 | £26 | £20 |
| Lifetime NMB (per person, £30,000 per QALY) | £594,362 | £594,396 | £594,406 | £594,403 | £594,393 | £34 | £44 | £41 | £31 |
| ICER (£ per QALY) | NA | NA | NA | NA | NA | £2,634 | £2,490 | £2,041 | £2,120 |
| Late Stage CRC (per 100,000 people) | 4182 | 4088 | 4065 | 4080 | 4103 | -93 | -116 | -101 | -79 |
| Late Stage CRC Incidence Reduction (%) | NA | NA | NA | NA | NA | 2.2% | 2.8% | 2.4% | 1.9% |
| Total CRC (per 100,000 people) | 6605 | 6520 | 6504 | 6507 | 6524 | -84 | -101 | -98 | -81 |
| Total CRC Incidence Reduction (%) | NA | NA | NA | NA | NA | 1.3% | 1.5% | 1.5% | 1.2% |
| CRC Mortality (per 100,000 people) | 3121 | 3055 | 3047 | 3055 | 3068 | -66 | -74 | -66 | -53 |
| CRC Mortality Reduction (%) | NA | NA | NA | NA | NA | 2.1% | 2.4% | 2.1% | 1.7% |
| FIT Invites (per person) | 6.72 | 7.64 | 7.57 | 7.61 | 7.65 | 0.92 | 0.85 | 0.90 | 0.93 |
| FIT Responses (per person) | 4.88 | 5.45 | 5.36 | 5.45 | 5.42 | 0.57 | 0.48 | 0.57 | 0.54 |
| Screening Colonoscopies (per 100,000) | 6855 | 7306 | 7497 | 7379 | 7218 | 451 | 641 | 523 | 363 |
| Increase in Colonoscopy Usage (%) | NA | NA | NA | NA | NA | 6.6% | 9.4% | 7.6% | 5.3% |
| QALY Quality Adjusted Life Year; NMB Net Monetary Benefit (calculated assuming a willingness to pay threshold of £20,000 or £30,000 per QALY); ICER Incremental Cost-effectiveness Ratio; CRC Colorectal Cancer; FIT Faecal Immunochemical Test; NA Not Applicable. | | | | | | | | | |

Supplementary Table 7: Full outcomes for scenario E: Alternative discount rates. Absolute and incremental average outcomes for cost-effectiveness, health benefits and resource use over the lifetime of each person in the English population at model start (age 30).

|  | **Absolute Values** | | | | | | **Incremental vs Comparator** | | | |
| --- | --- | --- | --- | --- | --- | --- | --- | --- | --- | --- |
|  | **Comparator DR 1.5%**  (all start age 60) | N**o Stratification DR 1.5%** (all start age 58) | **Sex Stratified DR 1.5%** (men start age 56, women start age 60) | **Comparator DR 5%**  (all start age 60) | N**o Stratification DR 5%** (all start age 58) | **Sex Stratified DR 5%** (men start age 56, women start age 60) | N**o Stratification DR 1.5%** (all start age 58) | **Sex Stratified DR 1.5%** (men start age 56, women start age 60) | N**o Stratification DR 5%** (all start age 58) | **Sex Stratified DR 5%** (men start age 56, women start age 60) |
| Lifetime Costs (£ per person, discounted) | £973.01 | £975.86 | £976.50 | £273.40 | £275.96 | £276.75 | £2.85 | £3.48 | £2.55 | £3.34 |
| Lifetime QALYs (per person, discounted) | 29.5707 | 29.5740 | 29.5749 | 15.5224 | 15.5230 | 15.5232 | 0.0033 | 0.0042 | 0.0006 | 0.0008 |
| Lifetime NMB (per person, £20,000 per QALY) | £590,440 | £590,503 | £590,522 | £310,174 | £310,184 | £310,188 | £63 | £81 | £10 | £13 |
| Lifetime NMB (per person, £30,000 per QALY) | £886,147 | £886,243 | £886,271 | £465,398 | £465,415 | £465,420 | £96 | £124 | £16 | £22 |
| ICER (£ per QALY) | NA | NA | NA | NA | NA | NA | £865 | £822 | £4,056 | £3,972 |
| Late Stage CRC (per 100,000 people) | 4182 | 4088 | 4060 | 4182 | 4088 | 4060 | -93 | -122 | -93 | -122 |
| Late Stage CRC Incidence Reduction (%) | NA | NA | NA | NA | NA | NA | 2.2% | 2.9% | 2.2% | 2.9% |
| Total CRC (per 100,000 people) | 6605 | 6520 | 6496 | 6605 | 6520 | 6496 | -84 | -109 | -84 | -109 |
| Total CRC Incidence Reduction (%) | NA | NA | NA | NA | NA | NA | 1.3% | 1.7% | 1.3% | 1.7% |
| CRC Mortality (per 100,000 people) | 3121 | 3055 | 3036 | 3121 | 3055 | 3036 | -66 | -85 | -66 | -85 |
| CRC Mortality Reduction (%) | NA | NA | NA | NA | NA | NA | 2.1% | 2.7% | 2.1% | 2.7% |
| FIT Invites (per person) | 6.72 | 7.64 | 7.60 | 6.72 | 7.64 | 7.60 | 0.92 | 0.88 | 0.92 | 0.88 |
| FIT Responses (per person) | 4.88 | 5.45 | 5.47 | 4.88 | 5.45 | 5.47 | 0.57 | 0.59 | 0.57 | 0.59 |
| Screening Colonoscopies (per 100,000) | 6855 | 7306 | 7491 | 6855 | 7306 | 7491 | 451 | 636 | 451 | 636 |
| Increase in Colonoscopy Usage (%) | NA | NA | NA | NA | NA | NA | 6.6% | 9.3% | 6.6% | 9.3% |
| QALY Quality Adjusted Life Year; NMB Net Monetary Benefit (calculated assuming a willingness to pay threshold of £20,000 or £30,000 per QALY); ICER Incremental Cost-effectiveness Ratio; CRC Colorectal Cancer; FIT Faecal Immunochemical Test; DR Discount Rate; NA Not Applicable. | | | | | | | | | | |
